# Awareness Level of Cancer Risk Factors and Warning Signs and Cancer Campaign Attendance behavior among Saudi Adults in a Tertiary Hospital in Riyadh

**DOI:** 10.31557/APJCP.2021.22.8.2421

**Published:** 2021-08

**Authors:** Essa M Sabi, Ahmed H A Mujamammi, Moath Abdulghani, Yasser M Almesfer, Anas A Alsuwaida, Abdullah Suliman Balobaid, Abdulaziz Fahad Alangari, Abdulrahman Saud Alharbi

**Affiliations:** 1 *Department of Pathology, Clinical Biochemistry Unit, Collage of Medicine , King Saud University, Riyadh, Saudi Arabia. *; 2 *College of Medicine, King Saud University, Riyadh, Saudi Arabia. *

**Keywords:** Cancer awareness, prevention, risk factors, symptoms of cancer

## Abstract

**Objective:**

To estimate the awareness level of Saudi adults about the risk factors and warning signs of cancer and observe the association of different determinants with cancer Knowledge in Riyadh city.

**Methods:**

A cross-sectional study carried out on 390 Saudis in the outpatient clinics of King Khalid University Hospital (KKUH), Riyadh. Data was collected using a validated Arabic questionnaire. Data was analyzed using SPSS software.

**Results:**

The overall awareness of the participants was limited, as the mean score of the overall cancer knowledge was 49.2%. Most of the correctly answered questions were on general cancer knowledge and not about risk factors or warnings signs. The only risk factors identified by most participants were smoking, alcohol, air pollution and genetic factors. Important risk factors such as physical inactivity, low dietary fibers and obesity were not well known. Despite Hepatitis B virus infection (HBV) being endemic in the kingdom, only 30% identified it as a risk factor. Apart from “A Thickening or a lump in breast or other organs”, < 50% of participants recognized alarming warning signs such as unexplained weight loss, unusual bleeding, and change in bowel habits. Females were more inclined to attend cancer awareness campaigns. Though people who reported attendance of cancer awareness campaigns surprisingly did not achieve a significantly higher overall knowledge score.

**Conclusion:**

The public lacks knowledge of well-established cancer risk factors and warning signs, despite recent advances in the medical field. Results suggest that current strategies to educate the public need to be revised.

## Introduction

Cancer is the second leading cause of death globally (WHO, 2018), and according to the International agency for research on cancer (IARC) was responsible for an estimated 10.0 million deaths in 2020 (Sung et al., 2021).In Saudi Arabia, the incidence of cancer is around 800 per 1,000,000 population per year and around 70% of cancer patients are diagnosed at an advanced stage (Sebai Zohair A., n.d.). In addition to the existing burden, the prevalence of cancer cases and deaths is expected to grow rapidly as populations grow older, and adopt lifestyle behaviors that increase the risk of cancer (Torre et al., 2016).

Fortunately, around 40–50% of cancers could be prevented if current knowledge about risk factors was translated into effective public health strategies (Stewart et al., 2016). These risk factors include tobacco products, alcohol consumption and not maintaining a healthy body weight or exercising regularly. Therefore, educating the public about such factors could potentially prevent more than one third of cancer deaths (Stewart et al., 2016).

In addition, it is possible to reduce the current burden of cancer with early detection, which can radically change the outcome of the disease for the better. To detect cancer in its less aggressive stages, one must be aware of early warning signs. The American Cancer Society has listed a few warning symptoms including: “*Thickening or lump, unexplained weight loss, change in a wart or mole, change in bowel habits or bladder function, unusual bleeding or discharge, nagging cough/hoarseness, and indigestion or trouble swallowing*” (“*Signs and Symptoms of Cancer | Do I Have Cancer*?,” n.d.). The problem is that the lack of awareness regarding key risk factors is greatly contributing to the high incidence of cancer (Stewart et al., 2016). Also, overlooking warning signs is resulting in late diagnosis and poor outcomes. (“*Signs and Symptoms of Cancer | Do I Have Cancer*?,” n.d.)

In Saudi Arabia, most of the previously conducted studies were outdated (Ravichandran et al., 2010) or targeted awareness of Individual cancers rather than an inclusive approach to multiple cancers (Al-Atif, 2021; Aljuhani et al., 2018; Al-Maweri et al., 2015; Almutairi et al., 2016; Alsareii et al., 2020; Galal et al., 2016; Musalli et al., 2021). Despite all the great efforts, a gap still need to be filled in order to initiate a comprehensive national cancer control plan which the present study attempts to fill. Almost a decade ago, it was reported that Saudis in Riyadh had poor awareness of cancer risk factors and warning signs (Ravichandran et al., 2010). During this period, the community has witnessed big changes in the accessibility to knowledge and technology.

In short, to decrease the occurrence of cancer, it is crucial that every individual in our population is aware that the lack of physical activity (Hardman, 2001), low fruit and vegetable intake (“*WHO | Promoting fruit and vegetable consumption around the world*,” n.d.), smoking, and other risk factors, are contributors to cancer development. Moreover, noticing a warning sign and going through cancer screening methods can improve prognosis immensely. While campaigns and other forms of awareness-raising programs are important and regularly conducted in Riyadh, this study aim to know who is attending these conventions, and whether or not they are benefiting from them. This study also aimed to assess the current efforts to educate the public and if the awareness level is influenced by different determinants (age, gender, attendance of previous campaigns).

## Materials and Methods

This is a quantitative, observational cross-sectional analytical study. This study was carried out in Riyadh city, Saudi Arabia. The data was collected over three months starting at August 2019. Data was obtained by self-administrated questionnaire that was answered by Saudi visitors and escorts in KKUH outpatient clinics in Riyadh city. The sample size was calculated using a single proportion sample size formula, n= z^2^p(1-p)/d^2^, with 95% confidence level and 5% Margin of error. The estimated sample size was about 385 chosen form 3,138,517 which is the number of Saudis in Riyadh city (General Authority for Statistics, 2015). To cover up for the anticipated uncompleted surveys, the sample size was increased to become 490, after filtering out uncompleted surveys and excluding those who did not fit the predetermined criteria (visitors from outside Riyadh, those younger than 18, those older than 65 and non-Saudis), 390 responses made it into the final analysis. To reduce selection bias, data was collected over alternating week days and alternating times of day over the span of three months. The validated questionnaire (Appendix A), which was adapted from a similar study (Qassim et al., 2018) was used. It was distributed in the form of paper-based survey to 20 individuals in a pilot study, and minor modifications were made to improve the clarity of the questions according to the respondents’ feedback. The questionnaire has been divided into four parts: the first contains demographic and general information about the participants; the second, is a group of general questions about cancer, the third contains questions related to cancer risk factors; and the fourth part, is a group of questions related to cancer warning signs. A question was included at the end of the survey to inquire about where the respondents had obtained their information. Informed consent was taken from participants before filling in the questionnaire. The participants’ rights to confidentiality were respected & no names or identification were included. No incentives or rewards were given to participants.

Data Analysis: Data was analyzed using SPSS software. The continuous measure variables were described in terms of mean and standard deviation, while independent variables were described in terms of frequency and percentages. To facilitate scoring of the knowledge test, answers were coded into binaries, where 1=correct answer and 0=incorrect/I don’t know. The Chi-square goodness-of-fit test was used to assess the difference in correct versus incorrect/not known answers for each question to test whether the majority of participants answered the question correctly or not and the statistical significance. The results are shown in Table 2 and discussed below. A total knowledge score was computed via adding up all of the 26 knowledge indicators after correcting them using the best correct answer key yielding a total knowledge score bounded between 0-52 maximum points, this knowledge score was re-scaled into percentage via dividing the total knowledge score by the maximum possible best correct cancer knowledge score (=26 items *X*^2^ marks each =52 maximum points/ marks) yielding a knowledge score expressed as a (%) out of hundred percent. To assess the impact of each independent variable on the score, accounting for other variables, a linear regression model was constructed to test the combined and individual effects of each variable on the knowledge score. As a secondary analysis, the Multivariate Binary Logistic Regression analysis was used to understand the pattern of people’s attendance or lack off to cancer awareness campaigns.

## Results

Three hundred and ninety Saudi residents had electively enrolled themselves into the study and completed and returned the survey, the yielded data analysis results of their sociodemographic characteristics are shown in Table 1. 59.5% of respondents were male. The sample was roughly evenly distributed across the four age groups, although most respondents were aged between 25-34 years. Regarding their geographic location, the majority (41.5%) were from North Riyadh, and only 6.2% resided in central Riyadh, while the remaining 53% were almost equally scattered across east, west and southern regions. The educational level of participants showed that most respondents had a high educational background, with 64.6% having a university education of some sort. The respondents were asked to indicate with (No/Yes) to whether they had previously attended any awareness campaigns for cancer screening and assessment, and 16.2% stated they have (Table 1).

*General cancer knowledge*

There was a significantly high number (87.9%; p<0.001) of Saudis that have a basic understanding about cancer in general, such as cancer being non-infectious in nature, and almost all (96.4%) participants appreciated the role of regular check-ups in early cancer detection. 

*Knowledge of cancer warning signs*

As for the knowledge of warning signs of cancer, only half of the participants identified unexplained loss of weight as a warning sign. Similar results were observed for unusual bleeding or discharge. Interestingly, the majority of participants were unable to identify many important warning signs, including difficulty swallowing, change in a wart or mole and a change in bowel or bladder habits (p<0.001). The only sign that was well-known to the people, was a thickening or lump in the breast or other organs (71%, p<0.001).

*Knowledge of cancer risk factors*

Unsurprisingly, 96.7% of respondents correctly agreed that smoking is a major cancer risk factor (p<0.001). Likewise, a significantly high proportion of Saudis correctly agreed that air pollution, alcohol consumption and genetic factors increase cancer risk. On the other hand, 91% of Saudis falsely believed that genetically modified foods were a risk factor (p<0.001). In line with the poor dietary awareness, significantly more (p=0.001) participants than expected by chance failed to recognize the increased cancer risk associated with low dietary fiber intake. Interestingly, 70% and 73% of respondents failed to identify HBV infection and aging as risk factors (p<0.001), respectively. Furthermore, there was no significant difference in the proportion of people who either did or did not correctly view obesity (p=0.07) and physical inactivity (p=0.12) as cancer risk factors.

Next, we sought to determine the overall scores of participants for each section of the questionnaire in Table 3, as well as to obtain an overall score for cancer knowledge across the entire questionnaire. The mean score for the general cancer knowledge section was ~8/10 (~80%), which indicates a good general knowledge about cancer. However, the mean knowledge of warning signs was with 6.5/18 points (36%), which denotes poor knowledge of warning signs. In addition, people scored ~11/24 (46.3%) on risk factor knowledge, which suggests low to moderate knowledge of cancer risk factors. Nonetheless, the overall knowledge score (total knowledge of all aspects) was 25.6/52 points on average, that is 49.2%, indicating that the participants have only a moderate overall knowledge about cancer. The Multivariate Linear Regression analysis, shown in Table 4, illustrated that participants’ age, gender, and employment status did not correlate significantly with their mean score, accounting for the other predictors in the analysis. On the other hand, educational level was significantly positively correlated with mean knowledge score: a higher educational level was a good indicator of a better score (p<0.001). Similarly, those associated with the medical field tended to score higher (p<0.001), compared to individuals not associated with the medical field, accounting for the other predictors. Surprisingly, attendance of cancer awareness campaigns did not significantly predict better cancer knowledge. In addition, the region of residence within Riyadh significantly affected the score (p=0.004). Further analysis showed that the west Riyadh in particular impacted the score.

*Cancer campaign Attendance Behavior*

The Multivariate Binary Logistic Regression analysis showed that the pattern of people’s attendance or lack off to cancer awareness campaigns was statistically significant, *χ*^2 ^(9)=25.64, p=0.002, denoting that at least one or more of the tested factors and covariates had a statistically significant association with people’s cancer campaign attendance behavior. The analysis showed that people’s age did not correlate significantly with their previous cancer campaign attendance, p=0.562, denoting that older and younger people had similar cancer awareness campaign attendance, by controlling for other predictors in the model. However, male participants were significantly less likely (O.R= 0.399 times less) to attend cancer awareness campaigns compared to females, p=0.003, by controlling for other predictors. In addition, the analysis model showed that people’s educational level and their occupation status did not correlate significantly with their cancer campaign attendance behavior, p>0.05 each. Notwithstanding, those associated with the medical field were 2.17 times more likely to have attended cancer awareness campaigns compared to those with no medical association, p=0.033, accounting for other predictors. Moreover, people’s general cancer knowledge subscore correlated significantly with their past cancer awareness campaign attendance. As people’s general cancer knowledge tended to rise by 1%, their mean predicted odds of attendance to cancer awareness campaigns increased by a factor equal to 22% times higher, or O.R=1.22 times higher, on average. On the other hand, people’s knowledge scores of cancer warning signs and risk factors did not correlate significantly with their odds of previous cancer campaign attendance, p>0.050 each respectively. Denoting that those people who previously attended and those who didn’t may not necessarily differ significantly on their mean knowledge scores of cancer risk factors and warning signs. Lastly but not least importantly, peoples residential location did not correlate significantly with their past cancer awareness campaign attendance behavior, p=0.539, controlling for other predictors.

**Table 1:** Descriptive Analysis of the Respondents Sociodemographic Characteristics. N=390

	Frequency (%)
Gender	
Female	158 (40.5)
Male	232 (59.5)
Age group	
18-24 Years	75 (19.2)
25-34 Years	115 (29.5)
35-44 Years	105 (26.9)
45-60 Years	95 (24.4)
Area within Riyadh	
East Riyadh	80 (20.5)
Middle	24 (6.2)
North Riyadh	162 (41.5)
South Riyadh	64 (16.4)
West Riyadh	60 (15.4)
Educational attainment level	
High school or less education	93 (23.8)
University	252 (64.6)
Postgraduate studies	45 (11.5)
Employment state	
Employee	242 (62.1)
Student	56 (14.4)
Unemployed	92 (23.6)
Association to the medical field	
No association	332 (85.1)
Student	23 (5.9)
Worker	35 (9)
Previously attended cancer awareness campaigns	
No	327 (83.8)
Yes	63 (16.2)

**Table 2:** Comparison of Indicators of General Cancer Knowledge, Knowledge of Early Warning Signs and Risk Factors of Cancer. N=390

	Incorrect answer/not known (%)	Correct answer (%)	p-value
General Knowledge of Cancer Risk			
Cancer is an infectious disease.	47 (12.1)	343 (87.9)	<0.001
A balanced diet reduces the possibility of getting cancer.	85 (21.8)	305 (78.2)	<0.001
Regular health checkup helps detect cancer early.	14 (3.6)	376 (96.4)	<0.001
Self-breast examination is one of the best ways to detect breast cancer early	144 (36.9)	246 (63.1)	<0.001
Regular exercise reduces the chances of getting cancer.	101 (25.9)	289 (74.1)	<0.001
Awareness of Warning Signs of Cancer			
Unexplained loss of weight.	198 (50.8)	192 (49.2)	0.76
A sore that does not heal.	289 (74.1)	101 (25.9)	<0.001
Unusual bleeding or discharge.	205 (52.6)	185 (47.4)	0.31
Thickening or lump in breast and other organs.	113 (29)	277 (71)	<0.001
Difficulty in swallowing.	269 (69)	121 (31)	<0.001
Nagging cough or hoarseness.	290 (74.4)	100 (25.6)	<0.001
Change in a wart or mole.	277 (71)	113 (29)	<0.001
Frequent urination or trouble urinating.	328 (84.1)	62 (15.9)	<0.001
Change in bowel habits.	275 (70.5)	115 (29.5)	<0.001
Knowledge Of Cancer Risk Factors			
Smoking	13 (3.3)	377 (96.7)	<0.001
Air pollution	151 (38.7)	239 (61.3)	<0.001
Genetically modified food	356 (91.3)	34 (8.7)	<0.001
Unbalanced diet (low fruit and vegetable intake)	227 (58.2)	163 (41.8)	0.0011
Alcohol consumption	155 (39.7)	235 (60.3)	<0.001
Infection by hepatitis B	274 (70.3)	116 (29.7)	<0.001
Being overweight or obese	272 (69.7)	118 (30.3)	0.077
Physical inactivity	210 (53.8)	180 (46.2)	0.128
Ionizing radiation (such as X-ray and CT scan)	180 (46.2)	210 (53.8)	0.128
Ultraviolet radiation exposure (sunlight)	233 (59.7)	157 (40.3)	<0.001
Genetic factors	162 (41.5)	228 (58.5)	<0.001
Old age	282 (72.3)	108 (27.7)	<0.001

**Table 3:** Descriptive Analysis of the Respondents Overall Measured Cancer Knowledge and Its Sub-Components

	Mean (SD)	Minimum-Maximum possible score
General Cancer Knowledge	7.99 (2.04)	0-10 points
Knowledge of Cancer warning signs	6.49 (5.01)	0-18 points
Knowledge of Cancer Risk Factors	11.1 (5.52)	0-24 points
Overall Knowledge of Cancer	25.59 (10.15)	0-52 points
Overall Knowledge of Cancer (%)	49.21 (19.51)	0-100 points

**Table 4:** Bivariate Analysis of the Respondents Overall Cancer Knowledge. N=390

	Mean (SD)-Overall knowledge (%)	test statistic	p-value
Gender			
Female	47.96 (18.70)	t(388)=0.50	0.620
Male	46.99 (18.88)		
Age group			
18-24 Years	48.10 (19.88)	f(3,386)=0.80	0.495
25-34Years	45.12 (17.95)		
35-44Years	48.32 (19.51)		
45-60Years	48.54 (18.14)		
Area within Riyadh			
East Riyadh	50.09 (18.39)	f(4,385)=3.10	0.016
Middle	49.07 (14.87)		
North Riyadh	48.38 (19.65)		
South Riyadh	47.97 (18.02)		
West Riaydh	39.81 (17.76)		
Educational attainment level			
High school	44.58 (20.76)	f(2,387)=6.92	0.001
University degree	49.44 (18.82)		
Higher/Postgraduate studies	57.52 (18.11)		
Employment state			
Employee	47.44 (18.49)	f(2,387)=2.44	0.088
Student	51.65 (20.57)		
Unemployed	44.65 (18.15)		
Medical professional			
No	45.69 (18.27)	t(388)=4.36	<0.001
Yes/student or workers	57.09 (18.91)		
Previously attended cancer awareness campaigns:			
No	46.39 (19.00)	t(388)=2.40	0.017
Yes	52.56 (16.85)		

**Table 5:** Multivariate Logistic Regression Analysis Explaining Peoples Attendance Behavior to Previous Cancer Awareness Campaigns. N=390

	Adjusted Odds Ratio	95% C.I.for Odds Ratio	
		Lower	Upper
Respondents Age group	0.922	0.7	1.214
Sex= Male	0.399	0.217	0.733
Educational Level	0.984	0.58	1.671
Occupation	0.964	0.672	1.382
Medical professional	2.167	1.063	4.418
General cancer Knowledge	1.22	1.025	1.452
Knowledge of cancer warning signs	1.032	0.967	1.103
Knowledge of cancer risk factors	0.987	0.928	1.05
Residential superb within Riyadh city	0.932	0.745	1.166
Constant	0.078		

Dependent variable, previous attendance to cancer awareness campaign (No/ Yes). Model predicted probability AUC ROC, 0.70. Reference group: Age group, 18-24; Sex, female; Education level, Highschool; Occupation, Employed; Medical professional, Non-medical professionals; General cancer knowledge, non attendants General cancer knowledge score; Knowledge of cancer warning signs, non attendants warning signs knowledge score; Knowledge of cancer risk factors, non attendants Risk factors score; Residential superb, East.

## Discussion

*General and overall cancer knowledge*

The study showed that the overall awareness of participants was limited, as the mean overall cancer knowledge score was only 49.2%. Furthermore, most of the correctly answered questions were in the general cancer knowledge section of the survey and not in the risk factors or warning signs. In contrast to a previous study from ten years ago on the same population (Riyadh citizens) (Ravichandran et al., 2010), it seems the overall score has decreased from 59.5% (Ravichandran et al., 2010) to now 49.2%. This decline may be due to the fact that a major source of information nowadays is social media which can be misleading and may propagate false information. This change can also be attributed to procedural differences between the two studies. However, 96.4% of participants appreciated the role of early detection in improving cancer outcomes. According to the previous study (Ravichandran et al., 2010), only 80.9% of participants were aware of this. The rise in awareness on this front is promising.

*Knowledge correlation with demographics*

Furthermore, our study has shown that age was not significantly associated with cancer knowledge, neither was gender. Perhaps this is due to the accessibility of medical knowledge online to all ages and genders. Notably, the attendance of previous cancer awareness campaigns did not predict significantly higher overall cancer knowledge. This could be because educational campaigns usually target individual cancers and don’t give a multidisciplinary view of cancers in general. Although, people who had previously attended campaigns measured slightly higher cancer knowledge than those who had not attended a campaign, the difference between them was not statistically significant, but this denotes possible practical importance. We recommend that a different approach or platform be utilized by the organizers of awareness campaigns in the future.

*Knowledge of cancer risk factors*

The average score was 46.3% on risk factor knowledge, suggesting low to moderate knowledge of cancer risk factors. The only risk factors identified by most participants were smoking, alcohol, air pollution and genetic factors. This reason for this might be that these factors get widespread media attention and coverage. Important risk factors such as physical inactivity, low dietary fibers and obesity were not known to most people. Unfamiliarity with factors related to diet could be connected to the adoption of Western food habits (which are high in fat and sugar), throughout the world. This is supported by similar findings in Oman (Al-Azri et al., 2014), Saudi Arabia (Ravichandran et al., 2010), Tanzania (Munishi et al., 2019), Sweden and Denmark (Lagerlund et al., 2015). In addition, it seems that there is a common misconception regarding genetically modified foods, as 91.3% of participants believed they increased cancer risk. This could be due to the spread of false information online.

Despite HBV infection being endemic in the kingdom (Abdo et al., 2012), only 30% identified it as a risk factor. This might hint as to why this disease continues to burden the kingdom’s health care system. Moreover, there was a worrying lack of knowledge towards aging (27.7%) as a risk factor which is concerning as the kingdom is aiming to increase the life expectancy to 80 as part of vision 2030 (Vision2030, 2017). This can reflect negatively on the motivation of Saudis to seek screening programs when indicated.

The majority of respondents identified smoking (96.7%) and alcohol consumption (60.3%) as cancer risk factors. This is consistent with the previous study on Riyadh residents in 2010 (Ravichandran et al., 2010), in which smoking (94.3%) and alcohol (80.4%) were well known cancer risk factors in the Saudi community. Also in Oman, Al-Azri et al. (Al-Azri et al., 2014) reported the same pattern and attributed the high knowledge of smoking (83.3%) and alcohol (69%) to the fact that these substances are forbidden in the Islamic faith and carry social stigma in this part of the world, this could explain the similar findings in the United Arab Emirates (Qassim et al., 2018). However this does not explain the results of a recent study in Hail (Alshammari et al., 2019), northern Saudi Arabia, which reported strongly conflicting results. In Hail, only 20.7% identified smoking as a risk factor and as little as 1.8% of participants seemed to agree that alcohol consumption increased cancer risk. A possible reason may be wide social acceptance of smoking in some Saudi Arabian suburbs, although alcohol is not socially accepted in the Arab world. Finally, when asked about UV light exposure, only 40.3% of participantsidentified it as a risk factor. This is consistent with findings elsewhere in the Gulf region (Al-Azri et al., 2014) which was attributed to the fact that skin cancer was uncommon in the Arabian Peninsula, due to the naturally protective skin pigmentation. On the other hand, in Australia, where skin cancer is much more common, 94.4% believed that sun tanning will increase the risk of cancer (Lizama et al., 2020). 

*Knowledge of warning signs*

Cancer warning signs were even less known when compared to risk factors. No warning signs were recognized by the majority of the population, except for thickening or a lump in the breast or other organs (71%). This was expected, as breast cancer is the most common cancer in Saudi Arabia (GLOBOCAN, 2018). On the other hand, signs of colorectal and thyroid cancer such as changes in bowel habits and hoarseness of voice, respectively, were barely recognized. This is alarming since these are the 2nd and 3rd most common cancers in Saudi Arabia, respectively (GLOBOCAN, 2018). These results share similarities with the study conducted in the United Arab Emirates (Qassim et al., 2018), where breast lumps were well recognized by university students as a cancer sign while changes in bowel habits was one of the least acknowledged signs. This might be due to bowel changes being a non-specific sign that most people experience in their lifetime.

Although 49.2% and 30% of Saudis recognized unexplained weight loss and change in a warts or mole as signs of cancer, respectively, these signs were even less recognized in the Indian subcontinent (Raj et al., 2012), which reported a much lower score. The difference between the two studies can be attributed to the larger sample size in India, although socio-economic discrepancies between the two countries can also play role.

*Cancer campaign attendance behavior*

The statistically significant convergence of general cancer knowledge on campaign attendance indicates that campaigns may have a significant effect on raising the general awareness of cancer but not necessarily the specific cancer risk factors and warning signs. This may be the result of the campaigns being focused on individual cancers and don’t give a multidisciplinary view of cancers in general as previously mentioned. The fact that females were more likely to attend Cancer awareness campaigns could be due to breast cancer being the most common cancer in the region, making it a leading public health concern. The efforts to educate women about screening and early detection have been enormous and many campaigns have targeted females specifically. Another reason for the disparities in attendance behavior between men and women could be due to shopping malls being a hotspot for educational campaigns in Saudi Arabia, and females are known to shop more often than males.

*Limitations*

One of the limitations of this study is the generalizability of the results to the population of Riyadh, this is because the respondents were not equally distributed across all geographic regions of the capital as the data was collected from the outpatient clinics of KKUH only. Another issue is the sampling technique, convenience sampling introduces selection bias, even though much effort was put forth in order to limit this effect by visiting outpatient clinics at different times of day on alternating days of the week. 

*Recommendations*

Despite recent advances in the field of medicine, the public still lacks knowledge of well-established cancer risk factors. Poor awareness of dietary risk factors such as low intake of fruits and vegetables seems to be almost a global trend, and the extent to which this relates to the high incidence of colorectal cancer needs to be investigated further. In Riyadh, Saudi Arabia, public understanding of cancer risk factors and warning signs was poor. Females formed the majority of the awareness campaigns attendees, and people who reported attendance of these campaigns did not score significantly higher. Indicating that perhaps the decision maker might want to reevaluate current strategies for public education.

## Author Contribution Statement

None.
